# Molecular Pathotyping of *Plasmodiophora brassicae*—Genomes, Marker Genes, and Obstacles

**DOI:** 10.3390/pathogens10030259

**Published:** 2021-02-24

**Authors:** Arne Schwelm, Jutta Ludwig-Müller

**Affiliations:** Institute of Botany, Technische Universität Dresden, 01062 Dresden, Germany; arne.schwelm@gmail.com

**Keywords:** brassica crops, clubroot, data sharing, effectors, genome, marker genes, pathotypes, *Plasmodiophora brassicae*

## Abstract

Here we review the usefulness of the currently available genomic information for the molecular identification of pathotypes. We focused on effector candidates and genes implied to be pathotype specific and tried to connect reported marker genes to *Plasmodiophora brassicae* genome information. The potentials for practical applications, current obstacles and future perspectives are discussed.

## 1. Introduction

Clubroot causes severe yield losses of brassica oil and vegetable crops world-wide [1]. The organism responsible for this disease is *Plasmodiophora brassicae.* Although originally referred to as a fungus, *P. brassicae* is a plasmodiophorid protist [2,3]. Plasmodiophorids contain chitin in the cell wall of their resting spores, but they are not related to fungi and unlike fungi or oomycetes do not show filamentous growth. Plasmodiophorids are part of the highly diverse eukaryotic group Rhizaria and belong to the Phytomyxea, a group of Endomyxa (Retaria) [3,4,5,6,7]. The Phytomyxea consist of obligate biotrophic parasites of brown algae, oomycetes (Phagomyxids) and diverse range of plant hosts (Plasmodiophorids) [3,6,8]. Due to the high agricultural and economically damage [1,9], *P. brassicae* is the best studied plasmodiophorid, although other species have a high impact on agriculture as well, such as *Spongospora subterranea* and *Polymyxa* species [2]. After the initial infection when zoospores encyst on the roots and inject themselves into the host cells, the root cortex is colonized. Plasmodiophorids are rare examples of plant pathogens that reside entirely inside their hosts where they multiply and form new resting spores [10,11]. Once re-released into the soil, *P. brassicae* can render infested fields unsuitable for brassica crop cultivation due to the persistence of resting spores in the soil for up to 20 years [12,13,14]. Chemical control for this soil borne disease is not possible at present and cultural practices, such as long crop rotation times, can only limit the soil infestation with *P. brassicae*. However, long crop rotation times are often not economical feasible especially for the cultivation of oilseed rape [9,13,14]. Therefore, the development of resistant cultivars is considered the most economical and efficient method for clubroot control [9,14]. Breeding for clubroot resistant plants has its own challenges, as it is work and time intensive, and resistance can be broken [15,16,17]. A deeper understanding of the molecular interaction between *P. brassicae* and its hosts, would facilitate the developing of new breeding and management strategies. Due to the truly intracellular lifestyle of *P. brassicae*, clubroot is a complicated system to study and research of this plant pathogen system lacks somewhat behind other plant–pathogen relationships. Most research has also been made using field isolates (*P. brassicae* resting spores collected from an infested plant or field) which might be heterogenic, and not with single spore isolates (SSI), a population derived from a host infected with a single spore. 

The first *P. brassicae* genome sequence from the European SSI e3 originally isolated from *Brassica rapa* was only published in 2015 (e3_2015) [18]. At the time, this was also only the third species of Rhizaria with genome information, which is one of the eukaryotic groups with the least molecular data [4]. By now genome drafts for the plasmodiophorids *Polymyxa betae* [19] and *Spongospora subterranea* [20] are also published. A further 48 *P. brassicae* genomes were assembled [21,22,23,24,25] and deposit in the NCBI genbank (https://www.ncbi.nlm.nih.gov/genome/browse/#!/eukaryotes/38756/ accessed on 10 November 2020). The genomes described in [24] were assembled based on the e3_2015 reference genome, whereas the other genomes described in this review were assembled de novo. The most recent assembly combined long- and short-read sequencing and accomplished a nearly complete assembly of the e3 genome (e3_2018). It consists of only 20 contigs (of which 13 are assembled chromosomes from telomere to telomere) with a total size of 25.1 Mb and also includes the complete mitochondrial sequence of 114 kbp in length [25]. The high quality of the e3_2018 genome will facilitate the reference-based genome assembly of additional *P. brassicae* isolates in the future. Assemblies in the NCBI database range between 24.05 and 25.25 Mb. The small size is due to a low presence of repeated sequences (2–5%) and a reduction of intergenic elements in the genome [18,21]. The number of protein coding genes is around 10,000, but gene models are only published for the e3 genomes. Many of the predicted proteins of *P. brassicae* do not show high similarities to protein models of other species or do not contain known functional domains, making the prediction of their function difficult [18,25,26].

As the protists cannot be cultivated without host, gene studies using reverse genetic methods (i.e. constructing of gene knock-out mutants) cannot be applied to study gene function. Thus the *P. brassicae* gene function remains for most cases hypothetical, despite the genome information. However, the genome information and transcription studies gave some insights into the pathogen metabolism (for a review see [27]). The clubroot pathogen appears to be dependent from host metabolites as the genome appears to be contain several incomplete metabolic pathways [18,21,22], a characteristic common with other eukaryotic biotrophic plant pathogens [28,29]. The missing genes encode proteins involved in sulfur and nitrogen uptake, and arginine, lysine, thiamine, and fatty acid biosynthesis pathways. In addition, only a few carbohydrate active enzymes (CAZymes), involved in the synthesis, metabolism, and transport of carbohydrates, were found. The *P. brassicae* genome contains genes potentially able to manipulate plant hormone metabolism, such as the auxin-responsive Gretchen Hagen 3, isopentenyl-transferases, a SABATH type methyltransferase and cytokinin oxidase [18,21,30]. The investigation of proteins associated with lipid droplet organelles [23] or protein families such as the E3 ubiquitin ligases of *P. brassicae* [31] or immunophilins [32] also benefitted from the available genome information. 

However, despite the presence of the genomes, most transcriptional studies just focus on the host response to an infection by *P. brassicae* and ignoring the information of the *P. brassicae* gene expression. Even though the *P. brassicae* gene expression pattern will be mainly descriptive, it contains important information. Even without functional domains encoded in the proteins, *P. brassicae* candidate genes can be selected from the transcript information for further studies to better understand how they manipulate the host and gives insights about how the metabolism of the pathogen changes [18,22,33,34,35,36]. Jiang et al. [35] did report differential expression of identified effector candidates in Canadian *P. brassicae* isolates 5I and 5X in resistant and susceptible *B. napus* hosts. Thus, the regulation of effector genes might lead to a host specific virulence of different isolates and should be investigated in the future. To identify differences in the gene regulation of effector candidates and other genes, analyses of the *P. brassicae* transcripts in more transcriptomic studies would be very helpful. That information should not be ignored, to better understand the clubroot disease and therefore also the resistance of the hosts. Additionally, it should be considered that the transcriptional host response is different in root tissue that is colonized by the pathogen than in *P. brassicae* free tissue [36] in a cell- and stage-specific manner [37], so that transcriptional analyses of whole roots contain diluted information about host response and pathogen gene expression.

## 2. Genomes and Pathotypes

The genomes sequences enable now comparative analyzes between different *P. brassicae* isolates. A comparative analyses of *P. brassicae* isolates is of high interest as *P. brassicae* exists in different pathotypes or races. The pathotypes are distinguished by the ability to infect different *Brassica* species or causing more severe disease symptoms and overcome resistance on certain *Brassica* hosts compared to other hosts. Knowing the pathotype that is present in the soil of a certain field, would be great advantage as farmer could chose to grow a crop variety which is less susceptible to the present *P. brassicae* isolate and thereby diminishing anticipated crop losses. To date the pathotype is determined by work and time intensive bioassays, which test the grade of infection on a set of hosts thereby identifying the ability of a *P. brassicae* isolate to infect different plant host genotypes harboring resistance genes. Currently different host sets are used internationally, such as the European Clubroot Differential system (ECD) [38], and pathotyping according to Somé [39] and Williams [40]. Additional adaptations were made using regional economical important hosts to fit local needs, such as the Canadian Clubroot differential set (CCD) with a focus on rapeseed resistance [41]. Other systems were focusing on Chinese cabbage resistance [42,43,44]. Those different systems make it difficult to compare pathotypes between studies, as the pathotype determined by one system cannot be translated into another system. However, the CCD system assigns *P. brassicae* isolates based on their Williams classification, along with a letter denoting their virulence pattern on the additional hosts of the CCD set and also includes the differential hosts of Somé [41].

Other systems focusing on Chinese cabbage resistance Different pathotypes occurring dominant in different regions or areas in the world. The Williams pathotype 3 appears to be dominant in Alberta, Canada [45,46], and Korea [43], whereas Williams pathotype 4 is dominant in China [42]. Using the ECD system dominant pathotypes were also determined in Australia as 16/3/12 and 16/3/31 [47]. In Germany, the *P. brassicae* isolates with the Somé pathoypes 1 and 3 or ECD pathotypes 16/31/31, 16/14/30, and 16/14/31 were most frequently found [17]. The occurrence of pathotypes is somewhat fluent and new pathotypes become present in fields, and there is variation of virulence inside a pathotype, when tested on additional hosts [15,42,43,44,46]. In addition, *P. brassicae* field isolates and even isolates from an individual plant root can consist out of a mixture of pathotypes and genetically different strains [48,49,50]. Thus, the homogeneity of the pathogen material can only be guaranteed if it has been multiplied from a single spore. Most pathotyping is performed with field isolates and the pathotype should be interpreted with caution. In the field, a less prominent *P. brassicae* pathotype might be present and become prominent, when a different host is cultivated.

Still, replacing the time-consuming bioassays by a fast and cheap molecular distinction between *P. brassicae* races, would be a huge advantage. Therefore, it must be known if and which sequence variations correlate with the race characteristics of the different isolates. A standardized pathotype system would therefore be beneficial to compare the molecular data from international isolates with each other. Furthermore, for isolates used in molecular studies a pathotype is often not determined. However, a large number of pathotyped isolates derived from different hosts and geographic origins is needed to identify molecular markers of different *P. brassicae* pathotypes and isolates. 

One obstacle for comparing the *P. brassicae* genome data with pathotype and other information is that many of the sequenced isolates have been named differently in different publications and again differently in the NCBI database. We summarized the currently available *P. brassicae* genome assemblies, linked with the information about origin, other assigned isolate names and pathotype (if known) in Table 1. The majority of the sequenced *P. brassicae* strains were isolated from canola and are of Canadian origin. Indeed, within the 43 genomes published recently, two originated in the USA, five from China, and the other sequences were obtained of Canadian isolates [24]. This study also included a number of SSIs and many of the isolates were pathotyped. The first reported Canadian *P. brassicae* genomes came from a variation of pathotypes [21]. The two genome assemblies deposited in the public databases derived from SSI of Williams pathotypes P3 (AAFC-SK-Pb3) and P6 (AAFC-SK-Pb6) whereas the first Chinese *P. brassicae* genome derived from SSI of Williams pathotype 1 (ZJ-1) [23]. Currently there are three genome assemblies of *P. brassicae* from Europe: the original sequence of the SSI e3 [18] and its updated version (e3_2018) [25] and the sequence of the selection isolate eH [22]. The eH isolate has a pathotype P1 according to Somé, but it is not SSI. However, both the isolate e3 and the isolate eH, originally derived from the same isolate “e” from a stubble turnip [51]. As the three European genome sequences come from *P. brassicae* isolated from the same clubroot, they do not allow a deeper insight into the genomic variation of European or even German *P. brassicae* isolates. RFLP analyses show a high genomic variation in European isolates [52,53], but to date genomic data are missing to analyze the variation in more detail. Currently additional European *P. brassicae* non-“e” sequences are only published from transcriptomes of clubroot infected kohlrabi (*Brassica oleracea* var. *gongylodes*) from Austria [36].

## 3. Gene Variation and Molecular Markers

Several studies tried to associate gene sequences to certain pathotypes or isolates. Comparisons of single nucleotide polymorphisms (SNPs) reveal differences in the genome assemblies of *P. brassicae* isolates [21,24]. A phylogeny based on SNPs of Canadian, Chinese and *P. brassicae* isolates from North Dakota (USA) in comparison to the e3_2015 sequence distinguished 5 different groups of *P. brassicae,* which however did not cluster according to their pathotypes [24]. Other studies looked at specific genes for their specificity of pathotypes. Polymorphism within the 28S rDNA of *P. brassicae* were reported which potentially could distinguish *P. brassicae* pathotypes, but unfortunately, the reported variation in LSU sequence of the rDNA of *P. brassicae* was due to chimeric PCR products of *P. brassicae* DNA and other soil inhabiting cercozoan species [60,61,62]. A set of markers was also reported to distinguish Korean isolates with different virulence patterns on clubroot resistant and susceptible cultivars of Chinese cabbage [63]. Markers were selected through sequence characterized amplified region (SCAR) by comparing the whole genome sequences of *P. brassicae* isolates from Korea with the genome of the e3_2015. However, while primer sequences were published the authors did not provide information about the sequence of the amplified regions or the genome assemblies used in their study, so the Korean sequences cannot be used in comparative studies.

Molecular markers were reported to distinguish the predominant Williams pathotypes P11, P9, P7 and P4 in China [64,65,66], as well as for P5 [67] and the new emerged pathotype P5X in Canada [68]. For now, it remains difficult to trust the reported PCR assays in [64,65,66]. Marker genes for the Williams P4 and P9 were identified using the e3_2015 genome and additional identified genes from transcriptome data, which were not predicted in the e3_2015 [64,65]. Unfortunately, the authors did not report the sequences of their new identified genes. It would be of interest to see if the sequences of the reported genes are present in the available *P. brassicae* genome assemblies and if those markers are useful for pathotype determination and indeed of *P. brassicae* origin. The authors reported further that the genes encoding for PBRA_003263, PBRA_003268, and PBRA_000003 can identify Williams pathotype P4. However, in the public available genome data the gene sequences for PBRA_000003 is present in all sequenced *P. brassicae* isolates without any sequence variation, inclusive Williams P1–P3, P5, P6, and P8 pathotypes. In contrast, PBRA_003263 and PBRA_003268 are missing in the AAFC-SK-Pb3 and AAFC-SK-Pb6 assemblies and PBRA_005772 is additionally not in part of the ZJ-1 assembly, but all are present in a variety of pathotypes. If those genes are only present in certain *P. brassicae* isolates or if the assemblies are incomplete needs to be tested. 

In a similar investigation by the same group PCR assays were reported to differentiate other pathotypes, using sequences of novel genes for which the *P. brassicae* origin was not confirmed, as well as the e3_2015 sequences for PBRA_007750, PBRA_008439, and PBRA_009348. From those genes PBRA_007750 and PBRA_008439 are partially present in the AAFC-SK-Pb6 assembly and present in all other genomes, albeit with sequence variations (Supplementary Data S1). There are no genome assemblies in NCBI databases with the Williams pathotype 4 or 7, but the primer pairs used to amplify the PBRA_007750 sequence would amplify the markers from Williams pathotypes 1–3, 5, 6, and 8 (Supplementary Data S1), but the reported distinction between Williams pathotypes 4 and 7 might be possible. PBRA_009348 is missing in AAFC-SK-Pb3 and has one nucleotide different in P.b-3 and P.b-17, and the sequence is otherwise identical in the genomes of the NCBI database. PBRA_000303, reported to be specific for pathotype P7 [66], is missing in the AAFC-SK-Pb3 and ZJ-1 genome assembly but present without sequence variation in all other genomes.

It might well be that the reported primers for the *P. brassicae* genes found in all genome sequences in the databases only amplify the genes in the reported Chinese isolates of a certain pathotype. Some reported primers do not appear to match the genome sequences in the NCBI database without mismatches and the primer sequences for PBRA_003263 published in [65] do not match the PBRA_003263 sequences obtained from the genome assemblies deposited in the NCBI database. The lack of information of the retrieved sequences of the Chinese isolates used in the studies above, do not allow to check sequence variations with other strains and pathotypes. The genes are however present in most if not all sequenced Williams pathotypes (P1–3, P5, P6, and P8) and it is therefore questionable if the reported PCR assays can be used to undoubtedly identify P4, P7, and P9. However as there are currently no genome assemblies of *P. brassicae* isolates from Williams P4, P7, and P9, so the reported markers might be able to differentiate between those pathotypes. 

The CR811 (KJ683723.1) gene was reported to be specific for the Canadian *P. brassicae* isolates of Williams pathotypes P5 and P5X [67]. However, the according CR811 sequence is not part of any of the published *P. brassicae* genome assemblies, including genome sequences for Canadian isolates of P5 and P5X; thus, it is either missing in the assemblies or not part of the *P. brassicae* genome and therefore not a specific marker. The origin of the CR811 gene should however be identified, as it could be that a higher virulence is associated with the presence of other microorganisms, which harbor this gene. 

Generally, a PCR assay to distinguish *P. brassicae* pathotypes does have additional obstacles. Pathotype diversity within single root galls appears to be a common occurrence. In a Canadian study 50 of 79 investigated galls consisted of more than one strain [48]. Therefore, the results using a single-gall or field population for pathotyping or molecular research, especially for the identification of pathotype specific markers, should be treated with caution. In a field and even in a single club several different isolates can be present [48,49]. While one isolate of a certain pathotype might be dominant, the PCR assay can still amplify DNA from the less present pathotypes. Additionally, it should also be shown that DNA derived from clubroots or soil can be amplified with a positive control, especially if a marker is supposed to be absent in certain pathotypes. It is likely that false positive or negative results from the PCRs will occur frequently. One solution might be a multiplex PCR assay. Yang et al [69] used two genes that were able to differentiate two groups of *P. brassicae* isolates via PCR. While this duplex PCR assay could differentiate between *P. brassicae* isolates that could break resistance on resistant canola cultivar 45H29 or not, the assay could also not determine pathotypes. The study also showed that field isolates are usually mixed population. In field isolates both specific bands were amplified and showed potential for quantitative analyses of different pathotypes in parallel [69].

## 4. Effectors

### 4.1. Effector Function

As for other plant pathogens, the identification of effector proteins is a focus for *P. brassicae* research [26]. Effectors are typically small-secreted proteins (SSP) often rich in cysteine residues. Plant pathogens use effectors to interfere with the host defense response or manipulate host cell processes for their own benefit [70].

The first verified effector protein of *P. brassicae*, a SABATH-type methyltransferase (PbBSMT), was identified even before the genome was sequenced [29]. Heterologous expression and enzymatic analyses, showed that PbBSMT can methylate salicylic acid (SA). The PbBSMT is able to convert SA to its more volatile methylated form (MeSA) thereby potentially removing the SA defense signal from the infected roots. In transcriptomic studies that analyzed *P. brassicae* genes PbBSMT is among the highest expressed genes during the infection indicating an important role in clubroot infections [18,36]. Indeed, does expression of the PbBSMT lower the defense potential of the host to other pathogens [71,72]. However, the draft genomes of the plasmodiophorid plant pathogens *Polymyxa betae* [19] and *Spongospora subterranea* [20], do not contain a PbBMST homolog, indicating that a PbBMST might not be essential for other plasmodiophorid species. As speculated before [27], fluorescence in situ hybridization suggests that PbBSMT is induced when the pathogen starts to produce spores [73] which might counteract the plant defense response when chitin, a potent inducer of plant defense responses [74], is produced. The suppression of SA-mediated defenses would facilitate *P. brassicae* to multiply in the host roots. Another potential effector candidate, the *P. brassicae* immunophilin-like protein PbCYP3, increased virulence on rice when heterologously expressed in a *Magnaporthe oryzae* gene-inactivated ΔCyp1 strain [32].

Effectors of oomycete pathogens often include the amino acid motif RXLR, which is believed to function in translocation of effector proteins into the plant host cells and similar to the PEXEL motif in the malaria pathogen *Plasmodium falciparum* [28,29]. However, potential RXLR and CRN are very rare in putative *P. brassicae* secreted proteins indicating that both motifs are not prevalent among the effectors of *P. brassicae* [18,21]. Other motifs enriched in *P. brassicae* effector candidates belonged to the functional domains of the chitin-binding CBM18 domain or the ankyrin domain, which are both functional domains of prominent proteins in the predicted *P. brassicae* secretome [18].

For *P. brassicae*, effector candidates were mainly identified by analyzing RNA-sequence data of infected hosts [18,21,22,33,34,75]. Based on the transcription pattern in different *P. brassicae* life stages of genes predicted to encode cysteine-rich small secreted proteins. They were grouped into predominantly plasmodial (PLeff), host connected (Heff) and late life cycle (Leff) effector candidates [18]. From RNA-sequence data of *P. brassicae* infected Arabidopsis roots, using a bioinformatics pipeline [34] and from transcript data of *P. brassicae* isolate from a rapeseed host [33] more effector candidates were predicted. In both studies the functionality of the effector candidates signalling peptides (SP) were tested using a yeast secretion assay. Among the candidates secreted in the yeast assay are proteins with predicted domains for ankyrins, kinases, proteases, and chitin binding (CBM) and Zinc finger domains, which are also found in effectors of other plant pathogenic organism. It was recently shown that in *Verticillium nonalfalfae* a CBM18-domain containing secreted protein acts as an effector by interfering with the chitin detection by host plant [76]. The genomes of *P. brassicae* and *S. subterranea* show an enrichment of proteins that include the chitin binding CBM18 domain, either as part of CE4-deacetylase proteins or as functional domains in SSP [18]. For two CBM18 domain containing SSPs of *P. brassicae* it indeed appears that they suppress chitin-triggered in *B. napus* [77]. 

The PBRA_008980 homologue SSPbP22 shows kinase activity and is localized to the cytoplasm and nucleus when expressed in plant cells and speculated to interfere with the host cell cycle [34], which is disturbed in clubroot tissue [78]. Kinase effectors are known from other plant pathogens, [79,80]. If this gene has an effector function for *P. brassicae* remains to be shown. 

The lack of functional domains and homologs in other organism in many of the predicted proteins is still an obstacle for the functional interpretation of *P. brassicae* genes. Ten of selected effector candidates by Chen et al. [33] did not contain functional domains. However, it was tested if they induce or suppress hypersensitive response in *B. napus* and tobacco. The hypothetical *P. brassicae* proteins PCBN_002550 and PCBN_05499 did induce necrosis in tobacco and in case of PBCN_002550 also in *B. napus*, when heterologous expressed in the plants. When effector candidates were co-expressed with PBCN_002550, the induced cell death was suppressed by 28 of the identified effector candidates and 24 effector candidates did suppress plant cell death induced by a mouse protein. Thus, the study delivered more evidence of effector functions of their candidates, showing that the heterologous expression system can be used to study *P. brassicae* effector candidates.

Five of the plasmodiophorid proteins that suppressed the induced cell death contained ankyrin repeat domains. Ankyrin repeats are very common protein–protein interaction motifs, frequently in secretomes of bacterial plant pathogens [81]. Bacteria use ankyrin domain containing to manipulate eukaryotic host functions [82]. In plants and ankyrin repeat-containing proteins can be involved in disease resistance, antioxidation metabolism, reactive oxygen production, and biotic and abiotic stresses [83], thus making secreted *P. brassicae* ankyrin proteins feasible effector candidates to interfere with the host metabolism.

### 4.2. Effector Variation and Pathotypes?

As effector proteins interfere with the host defense and metabolism, we hypothesize that these proteins are most likely those that responsible for making up different *P. brassicae* virulence characteristics, the pathotypes.

**Figure 1:**
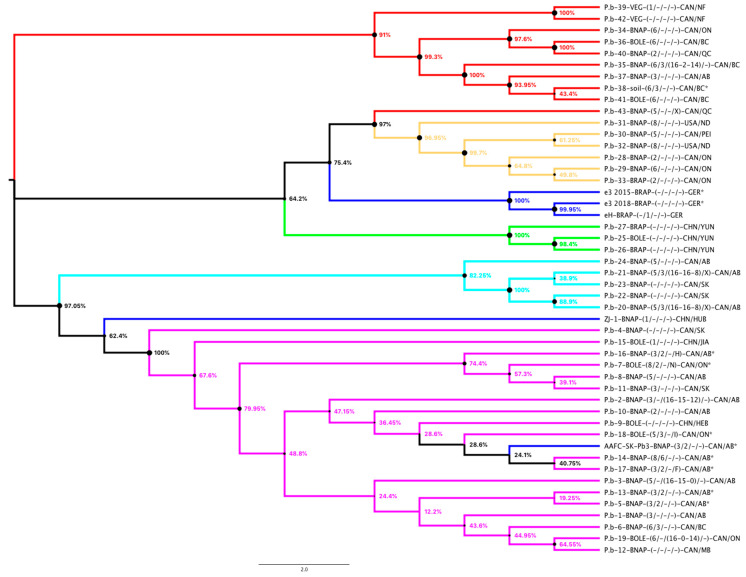
Phylogeny of *P. brassicae* isolates with a genome assembly (Table 1) using 26 effector candidates (Table 2). The color scheme of the isolates followed [24] (cluster 1= red; cluster 2 = yellow, cluster 3 = green, cluster 4 = light blue, cluster 5 = magenta, isolates not used in [24] are colored dark blue). The evolutionary history was inferred using the Minimum Evolution method using MEGA X [84]. The percentage of replicate trees in which the associated taxa clustered together in the bootstrap test (2000 replicates) are shown next to the branches. All ambiguous positions were removed for each sequence pair (pairwise deletion option) and a total of 21,376 positions in the final dataset.

Therefore, we attempted to build a phylogeny using effector candidates to check if we can obtain a pathotype specific clustering. We retrieved the genomic sequence from the *P. brassicae* genome assemblies (not including AAFC-SK-P6) of 26 effector candidates (indicated in bold in Table 2), to build an alignment and phylogeny (Figure 1). In this phylogeny we recovered the same clusters as determined in the SNP analyses in [24] with the exception of the P.b.-43 of pathotype 5X which grouped in cluster 2 instead of cluster 1. The “e”-isolates built their own group, whereas the Chinese ZJ-1 isolate is placed between cluster 4 and 5 of [24]. Thus, we could also not find a clustering according to the pathotypes of the sequenced *P. brassicae* isolates. The clusters 2, 3, and the cluster made of the “e”-isolates were the only clusters including *P. brassicae* isolates of *B. rapa*. The “e”-cluster is also a cluster of German isolates whereas cluster 3 is exclusive for Chinese isolates, but if this a true observation or due to limitation of samples, needs to be tested.

**Figure 2:**
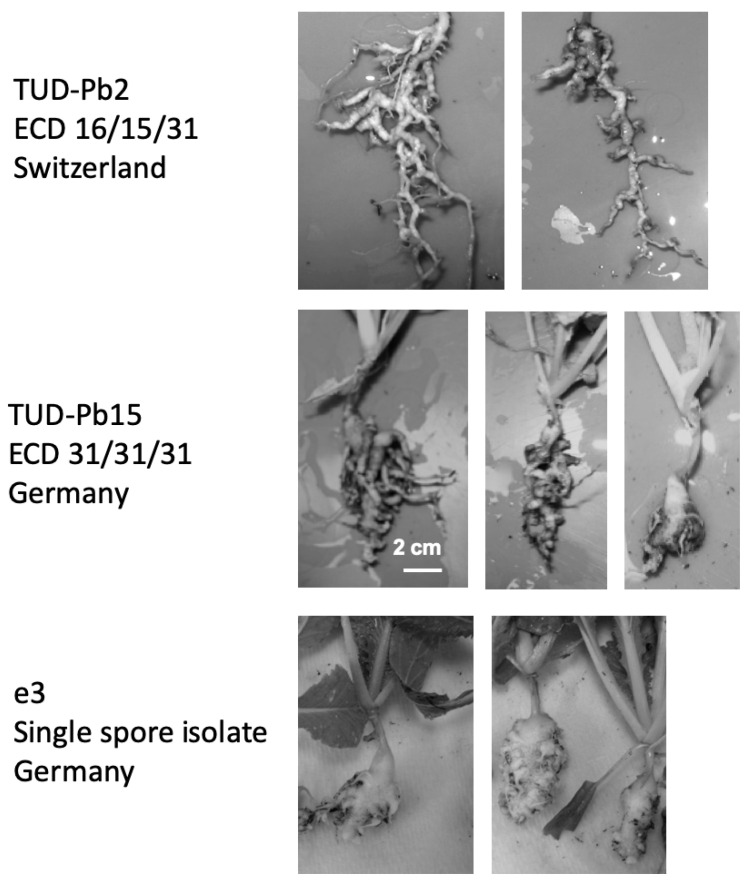
Typical galls of two selected Technische Universität Dresden (TUD) Pb isolates that are found in different clades of Figure 3 in comparison to the single spore isolate e3, all propagated on Chinese cabbage (*Brassica rapa* ssp. *pekinensis*). The bar indicates 2 cm for all photos.

**Figure 3:**
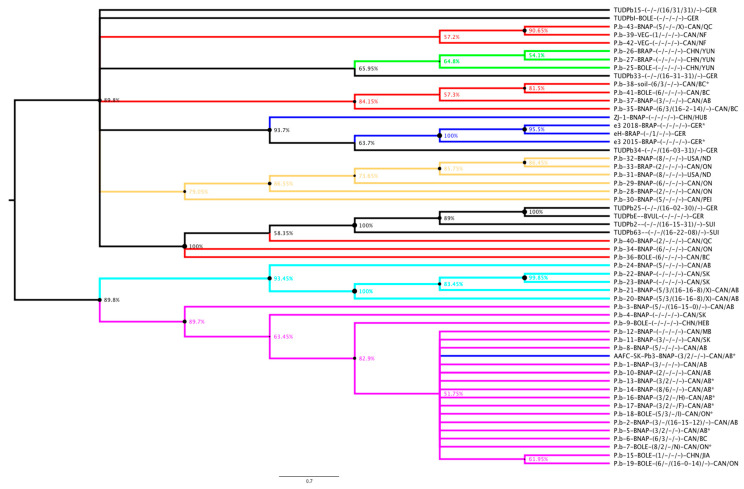
Phylogeny of *P. brassicae* isolates with a genome assembly (Table 1) and TUD isolates of Table 3 using the genome sequences of 5 effector candidates (PBRA_000619, PBRA_001191, PBRA_002462, PBRA_003620, and PBRA_005126). Sequences of TUD isolates were obtained by PCR amplification (Supplementary Table S1, Supplementary Data S3). The color scheme of the isolates followed [24] (cluster 1= red; cluster 2 = yellow, cluster 3 = green, cluster 4 = cyan, cluster 5 = magenta, isolates not used in [24] are colored dark blue, TUD isolates are colored in black). The evolutionary history was inferred using the Minimum Evolution method using MEGA X [84]. The alignments and complete sequences are given in Supplementary Datas S2 and S3.

To obtain a snapshot insight into the genetic variation of European *P. brassicae* isolates a subset of five effector candidate genes were PCR amplified from DNA of European isolates available at the Technische Universität Dresden (TUD) (Table 3). We amplified the gene sequence for the genes encoding effector candidates PBRA_000619, PBRA_001191, PBRA_002462, PBRA_003620, and PBRA_005126 from six German and two Swiss *P. brassicae* isolates (Table 3; Figure 2 and Figure 3) (Supplementary Datas S2 and S3). We constructed an additional phylogeny using those 5 effector gene candidates. In this analysis the *P. brassicae* sequences from the “e”-clubroot grouped closer together with the Chinese ZJ-1 isolate and TUDPb34. Based on this subset of genes a wider variation in the European *P. brassicae* isolates is indeed seen (Figure 3). The two isolates from Switzerland clustered together with the German isolates TUDPb25 and TUDPbE in a new built cluster. TUDPb15, TUDPbI, and TUDPb33 grouped in between Chinese and Canadian isolates from cluster 1 and 3 from the study of Bi et al. [23]. The snapshot phylogenies do suggest that the genetic variation of *P. brassicae* isolates might be more dependent on the geographical origin than on the pathotype. However, this observation is based on a very limited number of samples and genes and should be confirmed using whole genome comparative analyses with more isolates.

## 5. Conclusions

Through the availability of *P. brassicae* genomes the molecular investigation of the clubroot pathosystem has made huge progress. Effector candidates have been identified [18,21,33,34] and as methods are in place to characterize them in more detail [32,33,34,71,72,73] more detailed understanding of their function can be expected in the near future. However, in addition to the complications due to the *P. brassicae* obligate life style, a lack of analyses of *P. brassicae* genes, missing or difficult access to reported data, and the lack of genome data from a broad range of pathotyped isolates still restricts further progress by the research community. As *P. brassicae* is already a difficult system to work with we would like to urge the community to make all data accessible, so their efforts benefit the whole clubroot research community and validated by it. One fundamental question for applying molecular information into practice, is the issue of the genetic basis of *P. brassicae* pathotypes.

The molecular distinction between *P. brassicae* races in example through the detection of sequence variations, would be a huge advantage. If pathotype specific markers are identified, a fast and cheap detection system could replace the time-consuming bioassays. Therefore, it must be known if and which sequence variations correlate with the race characteristics of the different isolates. One obvious issue is the use of different pathosystems. It should be considered to develop a pathotyping system which is easy enough to handle to be applied by most *P. brassicae* researchers world-wide, to facilitate comparison of results and data. At least a common basic set of *Brassica* hosts should be included in locally adapted pathotyping systems to enable the interpretation of results internationally, as attempted by the CCD system [41]. Independent of which pathotype system is used, when marker genes are identified, their sequence and sequence variation should be reported so they can be investigated in other pathotyping systems and additional pathotypes. 

Whereas a molecular detection system through the presence or absence of a marker results in an unambiguous result, by its nature the current methods of pathotyping deliver a blurrier result. As in one field isolate more than one isolate or pathotype of *P. brassicae* can be present, the virulence pattern of the more dominant isolate will likely impact the pathotyping more than the less present pathotypes. A PCR-based molecular marker assay, however, will amplify the sequences of dominant as well as of the less present *P. brassicae* isolates of a population. Further the observed pathotype might be a consequence of a certain mixture of single pathotypes which might have an individually different virulence pattern than the tested population. Thus, if field or gall isolates of *P. brassicae* are used instead of single spore isolates, specific markers are only of limited use. Host plant material used in pathotyping is also not clonal, so each seed has a different genomic background. Homogenous plant material might be needed to clearly identify a pathotype. 

Our snapshot into the genetic variation of five genes in German and Swiss *P. brassicae* isolates showed that there is a much bigger variation in *P. brassicae* isolates than currently covered by the genome sequences. By sequencing more isolates an important knowledge gap could be closed. Genome assemblies are currently available of the Williams pathotypes P1-3, P5, P6, and P8, but studies from Asia often use involve the Williams pathotypes P4, P7, P9, and P11. Having genome assemblies of these pathotypes available would provide additional information needed to identify the pathotype specific characteristics. Ideally single spore isolates will be used for the determination of pathotypes to allow the detection of pathotype specific molecular markers. 

In our opinion, especially effector candidate sequence variations should be made accessible, as they are good candidates for host specificity of *P. brassicae* isolates or involved in resistance breaking. To identify more effector candidates, it is crucial that in transcriptomic clubroot investigations also the pathogen gene expression is analyzed. Certainly more effector candidates will come to light as seen in a recent study which identified a NUDIX-gene effector candidate in the eH isolate [75].

Variation in the effector repertoire and in the expression pattern and gene sequence might lead to molecular markers for pathotyping. In our analyses of effector candidate genes we did not see a grouping according to pathotypes of *P. brassicae* isolates. A similar result was reported by SNP analyses of 43 genomes (P.b-1 to P.b-43) [24]. However, we only looked at the sequence variation of effector candidates and differential regulation of effectors in the *P. brassicae* pathotypes [35] could be more important than sequence variation. When more studies will report and investigate the *P. brassicae* gene expression, regulatory networks of the pathogen should also become exposed. 

It might be a worthwhile community effort to sequence and assemble the genomes for single-spore isolate for each pathotype in one given pathotyping system, so there are standards for comparative analyses. To date there are already pathotyped genome assemblies from single spore isolates from Canada and China, but they only cover the Williams pathotypes P1, P3, P6, and P8 [21,24]. With more pathotyped single spore genomes, it might be possible to identify specific alleles and to develop markers that can identify the pathotype community of *P. brassicae* in samples from fields or galls. 

In 1975 Buczacki published his “Study of physiologic specialization in *Plasmodiophora brassicae*: Proposals for attempted rationalization through an international approach” [38]. Now the international community should attempt to develop a new international system by adapting the pathotype determination that the benefits of genome and transcriptome sequencing will facilitate the study the physiologic specialization in *P. brassicae*: A comparable pathotype system, genomes of pathotyped single spore isolates and fast and comprehensive access to data, and then, molecular markers may be used to gain a better understanding of the genetic variability and structuring factors within populations of *P. brassicae*.

## 6. Materials and Methods

### 6.1. DNA Extraction and PCR Primers

PCR-grade DNA was extracted using purified resting spores of *P. brassicae* isolates in Table 3 using a CTAB-based method [85] followed by DNA clean-up using the Genomic DNA Clean & Concentrator (Zymo Research) according to the manufacturer’s instructions. For primer and PCR conditions for amplifying the genes for Figure 3 see Supplementary Table S1. PCR products were purified and sequenced using Eurofins sequencing service. Gene sequences for the TUD isolates used in Figure 3 can be found in Supplementary Data S2 and the respective alignments in Data S3.

### 6.2. Phylogenies

For phylogenetic analyses, the selected effector candidate (Table 2) genomic sequences were retrieved from the NCBI genbank genomic sequence of the e3_2015 gene models. For each gene sequence a blastn search was performed using the whole-genome shotgun contigs (wgs) database of NCBI Blast limited to Plasmodiophorida (taxid:37357) organism. For each gene used in the phylogenies, the sequences were aligned using Bioedit [86] and Aliview [87] and manual curation. Alignments are provided in Supplementary Data S1. The phylogenic trees were constructed using MegaX [84].

## Figures and Tables

**Table 1:** Summary of available *P. brassicae* genome data.

Name in This Review Isolate-Host-(Williams/Some/ECD/CCD)-Origin	Isolate Name in NCBI Genbank (Other Names)	Origin	Host Origin	Bioproject Accession (NCBI)	Pathotypes				Single Spore
					Williams [38]	Somé [39]	ECD [40]	CCD [41]	
		**Canada**							
P.b-38-soil-(6/3/-/-)-CAN/BC *	P.b-38 (BC1-ss2-P6; AbotJE-ss2)	BC	soil	SAMN10755763	6	P3			SSI
P.b-6-BNAP-(6/3/-/-)-CAN/BC *	P.b-6 (BC2-ss4-P6; AbotJE-ss4)	BC	soil	SAMN10755731	6	P3			SSI
P.b-35-BNAP-(6/3/(16-2-14)/-)-CAN/BC	P.b-35 (BC3-P6; AbotJE-04-01)	BC	soil	SAMN10755760	6	P3	16/2/14		
P.b-36-BOLE-(6/-/-/-)-CAN/BC	P.b-36 (BC4-P6; P6)	BC	Brussels sprouts *(Brassica oleracea* var. *gemmifera)*	SAMN10755761	6				
P.b-41-BOLE-(6/-/-/-)-CAN/BC	P.b-41 (BC5-P6; P6)	BC	Cauliflower *(Brassica oleracea* var. *botrytis)*	SAMN10755766	6				
P.b-13-BNAP-(3/2/-/-)-CAN/AB *	P.b-13 (SCAN-ss1; AB1-P3)	AB	Canola *(Brassica napus)*	SAMN10755738	3	P2			SSI
P.b-16-BNAP-(3/2/-/H)-CAN/AB *	P.b-16 (SCAN-ss2; AB2-P3)	AB	Canola *(Brassica napus)*	SAMN10755741	3	P2		H	SSI
P.b-17-BNAP-(3/2/-/F)-CAN/AB *	P.b-17 (CAN-ss3; AB3-P2)	AB	Canola *(Brassica napus)*	SAMN10755742	2	P2		F	SSI
P.b-5-BNAP-(3/2/-/-)-CAN/AB *	P.b-5 (SCAN-ss4; AB4-P3)	AB	Canola *(Brassica napus)*	SAMN10755730	3	P2			SSI
P.b-14-BNAP-(8/6/-/-)-CAN/AB *	P.b-14 (CDCN-ss1; AB6-P8)	AB	Canola *(Brassica napus)*	SAMN10755739	8	P6			SSI
P.b-3-BNAP-(5/-/(16-15-0)/-)-CAN/AB	P.b-3 (CDCN-04-01; AB7)	AB	Canola *(Brassica napus)*	SAMN10755728	5		16/15/0		
P.b-10-BNAP-(2/-/-/-)-CAN/AB	P.b-10 (F-1-05; AB8-P2)	AB	Canola *(Brassica napus*	SAMN10755735	2				
P.b-24-BNAP-(5/-/-/-)-CAN/AB	P.b-24 (F290-07; AB9)	AB	Canola *(Brassica napus)*	SAMN10755749	5				
P.b-1-BNAP-(3/-/-/-)-CAN/AB	P.b-1 (AB10-P3)	AB	Canola *(Brassica napus)*	SAMN10755726	3				
P.b-37-BNAP-(3/-/-/-)-CAN/AB	P.b-37 (Deora; AB11- P3)	AB	Canola *(Brassica napus)*	SAMN10755762	3				
P.b-8-BNAP-(5/-/-/-)-CAN/AB	P.b-8 (Deora; AB12- P5)	AB	Canola *(Brassica napus)*	SAMN10755733	5				
P.b-21-BNAP-(5/3/(16-16-8)/X)-CAN/AB	P.b-21 (LG1; AB13-P5X)	AB	Canola *(Brassica napus)*	SAMN10755746	5	P3	16/6/8	X	
P.b-20-BNAP-(5/3/(16-16-8)/X)-CAN/AB	P.b-20 (LG3; AB14-P5X)	AB	Canola *Brassica napus*	SAMN10755745	5	P3	16/6/8	X	
P.b-2-BNAP-(3/-/(16-15-12)/-)-CAN/AB	P.b-2 (SCAN-03-01; AB15- P3)	AB	Canola *(Brassica napus)*	SAMN10755727	3		16/15/12		
P.b-11-BNAP-(3/-/-/-)-CAN/SK	P.b-11 (SK1-P3)	SK	Canola *(Brassica napus)*	SAMN10755736	3				
P.b-4-BNAP-(-/-/-/-)-CAN/SK	P.b-4 (CD1A; SK2)	SK	Canola *(Brassica napus)*	SAMN10755729					
P.b-22-BNAP-(-/-/-/-)-CAN/SK	P.b-22 (SK3)	SK	Canola *(Brassica napus)*	SAMN10755747					
P.b-23-BNAP-(-/-/-/-)-CAN/SK	P.b-23 (SK3)	SK	Canola *(Brassica napus)*	SAMN10755748					
P.b-12-BNAP-(-/-/-/-)-CAN/MB	P.b-12 (MB)	MB	Canola *(Brassica napus)*	SAMN10755737					
P.b-7-BOLE-(8/2/-/N)-CAN/ON *	P.b-7 (ORCA-ss2; ON1- P8)	ON	Cabbage *(Brassica oleracea* L. var. *capitata)*	SAMN10755732	8	P2		N	SSI
P.b-18-BOLE-(5/3/-/I)-CAN/ON *	P.b-18 (ORCA-ss3; ON2- P5)	ON	Cabbage *(Brassica oleracea* L. var. *capitata)*	SAMN10755743	5	P3		I	SSI
P.b-19-BOLE-(6/-/(16-0-14)/-)-CAN/ON	P.b-19 (ORCA.04; ON3)	ON	Cabbage *(Brassica oleracea* L. var. *capitata)*	SAMN10755744	6		16/0/14		
P.b-29-BNAP-(6/-/-/-)-CAN/ON	P.b-29 (ON4-P6)	ON	Canola *(Brassica napus)*	SAMN10755754	6				
P.b-28-BNAP-(2/-/-/-)-CAN/ON	P.b-28 (ON5-P2)	ON	Canola *(Brassica napus)*	SAMN10755753	2				
P.b-34-BNAP-(6/-/-/-)-CAN/ON	P.b-34 (ON6-P6)	ON	Canola *(Brassica napus)*	SAMN10755759	6				
P.b-33-BRAP-(2/-/-/-)-CAN/ON	P.b-33 (ON7- P2)	ON	Canola/ Pak Choi *Brassica rapa (Brassica napus)*	SAMN10755758	2				
P.b-43-BNAP-(5/-/-/X)-CAN/QC	P.b-43 (QC1- P5X)	QC	Canola *(Brassica napus)*	SAMN10755768	5			X	
P.b-40-BNAP-(2/-/-/-)-CAN/QC	P.b-40 (QC2- P2)	QC	Canola *(Brassica napus)*	SAMN10755765	2				
P.b-30-BNAP-(5/-/-/-)-CAN/PEI	P.b-30 (PEI1-P5)	PEI	Canola *(Brassica napus)*	SAMN10755755	5				
P.b-42-VEG-(-/-/-/-)-CAN/NF	P.b-42 (DD1- NF1)	NF	Vegetable	SAMN10755767					
P.b-39-VEG-(1/-/-/-)-CAN/NF	P.b-39 (DD2A; NF2-P1)	NF	Vegetable	SAMN10755764	1				
AAFC-SK-Pb3-BNAP-(3/2/-/-)-CAN/AB *	AAFC-SK-Pb3 (Pb3; SACAN-ss1)	AB	Canola *(Brassica napus)*	SAMN06010517	3	P2			SSI
AAFC-SK-Pb6-BNAP-(6/-/-/M)-CAN/AB *	AAFC-SK-Pb6 (Pb6; AbotJE-ss1)	BC	Vegetable soil	SAMN10342669	6			M	SSI
		**USA**							
P.b-31-BNAP-(8/-/-/-)-USA/ND	P.b-31 (ND1-P8; NDCR1)	ND	Canola *(Brassica napus)*	SAMN10755756	8				
P.b-32-BNAP-(8/-/-/-)-USA/ND	P.b-32 (ND2-P8; NDCR2)	ND	Canola *(Brassica napus)*	SAMN10755757	8				
		**China**							
P.b-15-BOLE-(1/-/-/-)-CHN/JIA	P.b-15 (CH1-P1)	Jiangsu, Ganyu	Kai-lan *(Brassica oleracea* var. *alboglabra)*	SAMN10755740	1				
P.b-26-BRAP-(-/-/-/-)-CHN/YUN	P.b-26 (CH2)	Yunnan, Muding	Chinese cabbage *(Brassica rapa* L. subsp. *Pekinensis)*	SAMN10755751					
P.b-27-BRAP-(-/-/-/-)-CHN/YUN	P.b-27 (CH3)	Yunnan, Muding	Chinese cabbage *(Brassica rapa* L. subsp. *Pekinensis)*	SAMN10755752					
P.b-25-BOLE-(-/-/-/-)-CHN/YUN	P.b-25 (CH4)	Yunnan, Lufong	Cabbage *(Brassica oleracea* L. var. *capitata)*	SAMN10755750					
P.b-9-BOLE-(-/-/-/-)-CHN/HEB	P.b-9 (CH5)	Hebei, Kuyuang	Broccoli *(Brassica oleracea* var. *italica)*	SAMN10755734					
ZJ-1-BNAP-(1/-/-/-)-CHN/HUB *	ZJ-1	Hubei	Canola *(Brassica napus)*	SAMN05440575	1				SSI
		**Germany**							
e3_2015-BRAP-(-/-/-/-)-GER *	e3		stubble turnip *(Brassica rapa* subsp. *rapa)*	SAMEA3232990					SSI
e3_2018-BRAP-(-/-/-/-)-GER *	e3		stubble turnip *(Brassica rapa* subsp. *rapa)*	SAMEA104666271					SSI
eH-BRAP-(-/1/-/-)-GER	eH		stubble turnip *(Brassica rapa* subsp. *rapa)*	SAMN08196759		P1			

The country and region of origin, the host the isolate derived from, alternative names, and pathotyping results have been retrieved from available literature [15,18,21,22,23,24,25,45,54,55,56,57,58,59] and information provided by the authors from [24]. Abbreviations: AB: Alberta, BC: British Columbia: PEI; Prince Edward Island, ON: Ontario, MB: Manitoba, QC: Quebec, CHN: China, USA: United States of America, ND: North Dakota, SK: Saskatoon, GER: Germany, CAN: Canada, BRAP: *Brassica rapa*; BNAP: *B. napus*; BOLE: *B. oleracea*; VEG: vegetable, EDC: European Clubroot Differential; CCD: Canadian Clubroot Differential. The numbers and letters in the columns for the spore classifications are based on those in the original publications. An * indicates single-spore isolates (SSI).

**Table 2:** *P. brassicae* effector candidates used in phylogenies.

Effector Candidate							Effector Candidate Characteristics			
Genbank Accession	e3_2015 Protein Name	Expressed in Clubroots [36]	Effector Candidate [33]	Effector Candidate [34]	Amplified from TUD Isolates	Effector Candidate [18]	Secreted in Yeast Assay [33,34]	Domains	Introns	Present in All Genomes
CEP03656.1	PBRA_003263						n.d.	-	0	Partial in AAFC-SK-Pb6
CEP00016.1	PBRA_007750						n.d.	DNA_BRE_C	6	Not in AAFC-SK- Pb6
CEO97274.1	PBRA_000619		PBCN_000619		YES		YES	-	0	YES
CEO97388.1	PBRA_000733		PBCN_000733			Heff	YES	recA		
CEO97459.1	PBRA_000804			SSPbP01			YES		0	YES
CEO99285.1	PBRA_001191	YES	PBCN_001191		YES		YES	-		
CEP01250.1	PBRA_001856		PBCN_001856	SSPbP18		Pleff	YES	-	0	YES
CEP01381.1	PBRA_001987		PBCN_001987				YES	zf-MYND	0	YES
CEP02197.1	PBRA_002462		PBCN_002462		YES	PLeff	YES	-	0	YES
CEP02583.1	PBRA_002550		PBCN_002550			PLeff	YES	-	0	YES
CEP02651.1	PBRA_002618			SSPbP03			YES	GlpG	0	YES
CEP03198.1	PBRA_002958		PBCN_002958				YES	ChtBD1	0	YES
CEO95618.1	PBRA_004344		PBCN_004344				YES	-	0	YES
CEO95633.1	PBRA_004359			SSPb11P			YES	PLN02633	3	YES
CEO96073.1	PBRA_004763	YES	PBCN_004763				YES	-	0	Partial in AAFC-SK-Pb6
CEO96517.1	PBRA_005126	YES	PBCN_005126		YES		YES	vWFA	0	YES
CEO96836.1	PBRA_005440		PBCN_005440	SSPbP31			NO/YES	-	0	Partial in AAFC-SK-Pb6
CEO96852.1	PBRA_005456		PBCN_005456				YES	Ank_2	0	YES
CEO97087.1	PBRA_005691		PBCN_005691				NO	-	1	YES
CEO97112.1	PBRA_005716			SSPbP44			YES			
CEO97859.1	PBRA_005973	YES		SSPbP04			YES			
CEO98671.1	PBRA_006785		PBCN_006785				YES	eIF3_subunit	1	YES
CEP00097.1	PBRA_007831	YES		SSPbP10			YES	-	0	
CEP03502.1	PBRA_009387	YES	PBCN_009387				NO	Ribosomal_L22	0	YES
CEO95090.1	PBRA_009622		PBCN_009622				YES	-	0	YES
CEO98583.1	PBRA_006697		PBCN_006697				YES	ANK	1	Not in AAFC-SK- Pb6; partial in AAFC-SK-Pb3

TUD: Technische Universität Dresden; (-): no defined protein domain.

**Table 3:** *P. brassicae* field isolates used in the five gene phylogeny in Figure 3.

Isolate	Origin	Original Host	ECD Pathotype	Name in Phylogeny (Figure 3)
TUD-Pb2	Switzerland	unknown	16/15/31	TUDPb2-(-/-/(16-15-31)/-)-SUI
TUD-Pb15	Berlin, Germany	unknown	16/31/31	TUDPb15-(-/-/(16/31/31)/-)-GER
TUD-Pb25	Leipzig, Germany	unknown	16/02/30	TUDPb25-(-/-/(16-02-30)/-)-GER
TUD-Pb33	Nordrhein-Westfalen, Germany	unknown	16/31/31	TUDPb33-(-/-/(16-31-31)/-)-GER
TUD-Pb34	Rheinland-Pfalz, Germany	unknown	16/03/31	TUDPb34-(-/-/(16-03-31)/-)-GER
TUD-Pb63	Switzerland	unknown	16/22/08	TUDPb63-(-/-/(16-22-08)/-)-SUI
TUD-PbE	Germany	Sugar beet ^1^ (*Beta vulgaris*)	unknown	TUDPbE-BVUL-(-/-/-/-)-GER
TUD-PbI	Frankfurt, Germany	Savoy cabbage (*Brassica oleracea* convar. *capitata* var. *sabauda*)	unknown	TUDPbI-BOLE-(-/-/-/-)-GER

^1^ origin from sugar beet root, but amplified on Chinese cabbage (*Brassica rapa* ssp. *pekinensis*).

## Supplementary Materials

The following are available online at https://www.mdpi.com/2076-0817/10/3/259/s1, Data S1: Alignment of 26 genes used for Figure 1, Data S2: Fasta file of alignment of gene sequences amplified by PCR from TUD isolates, Data S3: Sequences of 6 genes used in Figure 3, Table S1: PCR primers and conditions used for the amplification of effector gene sequences from TUD isolates.
