# Supplementary material for: Molecular Pathotyping of Plasmodiophora brassicae—Genomes, Marker Genes, and Obstacles
Source: Pathogens. 2021 Feb 24;10(3):259. doi: 10.3390/pathogens10030259 (PMC7996130; doi:10.3390/pathogens10030259)
Supplement: Supplementary file 1 [file pathogens-10-00259-s001.zip › supplementary files/Table S1.docx]

**Table S1.** Primer sequences and amplification conditions for PCR products used for the phylogeny in Figure 3. Complete sequences are shown in Data S3.

| **Primer name** | **Sequence** | **Condition** |
| --- | --- | --- |
| 001191-full-F  001191-full-R | CGCCCTTGCGAATGCC  GGCCGAACCTGAATTCGTC | Initial 94°C 2 min  35x:  94°C 20 sec  Annealing (55 or 59°C) 20 sec  72°C 45 sec  Final 72°C 3 min |
| 002462full-R  002462-full-F | CGAAAAGTGTGCCTGTAGAC  GCGTCATTCGTGATTTCTACTG |  |
| 000691-full-F  000691-full_R | GTGACGTCGTCGGCTG  GTCGGGTCGAACATGAACG |  |
| 005126-full-F  005126-full-R | GCCATGCCAAGTCCGATC  GCAGAGCGCGTGGTG |  |
| 003620-full-R  003620-full-F | TATCGATCGGCAAGGGACC  TCGCACATTGATCGAGTCG |  |
